# A Cancer Education Framework for Australian Medical Schools: an Announcement of a New Educational Program

**DOI:** 10.1007/s13187-022-02173-9

**Published:** 2022-06-28

**Authors:** Darren L. Starmer, Kylie Russell, Dianne Juliff

**Affiliations:** 1 School of Medicine, Faculty of Medicine, Nursing and Midwifery, Physiotherapy and Health Sciences, The University of Notre Dame Australia, 32 Mouat St, Fremantle, WA 6160 Australia; 2 School of Nursing and Midwifery, Faculty of Medicine, Nursing and Midwifery, Physiotherapy and Health Sciences, The University of Notre Dame Australia, 32 Mouat St, Fremantle, WA 6160 Australia

**Keywords:** Cancer education, Medical curriculum, Curriculum development, Expert review

## Abstract

This framework draws upon national and international cancer curricula to identify the essential cancer-related learning outcomes for Australian medical students. The framework incorporates feedback from medical, radiation and surgical oncologists, haematologists, and palliative care physicians on what medical graduates need to know about cancer. The consensus view was that medical students require a basic understanding of the principles of cancer management and the opportunity to see cancer patients in a cancer service unit. The framework assumes that certain knowledge, skills, and attitudes are already embedded in current Australian medical school curricula, presenting instead only the core cancer content in order to provide a clear and concise framework designed to maximise integration within existing curricula.

**Supplementary Information:**

The online version contains supplementary material available at 10.1007/s13187-022-02173-9.

## Aim

To develop a framework to assist medical schools to incorporate cancer education into an existing medical curriculum. The framework aims to provide a minimal set of clinical experiences and learning outcomes, which, if resourced, will provide Australian medical students with a basic understanding of the knowledge and principles underpinning current cancer management.

## Background

Currently, half of all Australians will be diagnosed with a cancer by the age of 85 [1, 2]. Whilst cancer is the leading cause of death in Australia [3], increased survivorship places significant demands on the health system [4]. Studies highlight that Australian medical students are ill-prepared to care for cancer patients upon graduation [5–8]. Additionally, medical students and junior doctors have questioned the quality of their own cancer education [8–12]. The lack of a national medical curriculum results in considerable diversity in cancer teaching and clinical exposure between schools [5, 13]. Nationally, both oncology [14, 15] and palliative care curricula have been developed [16]. However, their influence on Australian medical school curricula remains largely unknown [8, 10].

## Development

Consensus development panel evaluation of the Ideal Oncology Curriculum for Australian Medical Schools [15], combined with a review of the current literature on cancer and palliative care education, was undertaken to identify common themes and key areas appropriate for medical students. A draft framework was developed and reviewed by three cancer clinicians, prior to being reviewed by both national and international medical, radiation and surgical oncologists, haematologists, palliative care physicians, and general practitioners. It was agreed that medical students require a fundamental understanding of the principles of cancer management, coupled with exposure to cancer patients in cancer service units. Similarly, there was agreement that medical students do not require specialist knowledge, such as drug or radiotherapy dosages.

Knowledge that would be expected in all Australian medical schools (e.g. concepts of incidence and mortality, and evidence-based practice) were excluded to reduce the size of the framework and to optimise its utility and adoption. The framework draws heavily from the Cancer Council Australia’s Ideal Oncology Curriculum for Medical Schools [15], and the aforementioned review by cancer clinicians [17]. Other curricula used in the development of this framework include the Palliative Care Curriculum for Undergraduates [16] and the International Summer School ‘Oncology for Medical Students’ curriculum [18]. Although designed for the Australian medical schools, an international review of the framework highlighted its applicability to international contexts.

The framework (see below) is comprised of three sections: one focusing on clinical exposure to cancer patients and clinical cancer service units, whilst the other two focus on the principles of cancer management and cancer-specific knowledge. Suggested resources have also been included in the framework, to assist in supporting student learning and which would facilitate the inclusion of the framework as an extracurricular learning opportunity. The resources provide a good starting point and are freely available on the Internet, making them accessible to all schools and students. The full version of the framework document is provided as a supplementary document.

## A Cancer Education Framework for Australian Medical Schools



## Conclusion

The cancer education framework for Australian medical schools presents a minimal blueprint from which medical schools can develop a basic cancer curriculum within an existing curriculum. The content comprises the key aspects of cancer and palliative care education presented in the current literature and has undergone both national and international review to ensure that the content is relevant to medical graduates regardless of their chosen career path.

## Supplementary Information

Below is the link to the electronic supplementary material.

Supplementary file1 (PDF 481 KB)
