# Supplementary material for: A Cancer Education Framework for Australian Medical Schools: an Announcement of a New Educational Program
Source: J Cancer Educ. 2022 Jun 28;38(2):677–81. doi: 10.1007/s13187-022-02173-9 (PMC10102091; doi:10.1007/s13187-022-02173-9)

CANCER EDUCATION FRAMEWORK FOR AUSTRALIAN MEDICAL SCHOOLS

Abstract

This framework draws upon national and international cancer curricula to identify the essential cancer-related learning outcomes for Australian medical students. The framework incorporates feedback from medical, radiation and surgical oncologists, hematologists and palliative care physicians on what medical graduates need to know about cancer. The consensus view was that medical students require a basic understanding of the principles of cancer management and the opportunity to see cancer patients in a cancer service unit. The framework assumes that certain knowledge, skills and attitudes are already embedded in current Australian medical school curricula, presenting instead only the core cancer content in order to provide a clear and concise framework designed to maximize integration within existing curricula.

Darren Starmer

darren.starmer@nd.edu.au

Cancer Education Framework for Australian Medical Schools

Framework

This Framework has been created for the use of Australian Medical Schools.

Copy writed ©2020 all rights reserved

Authors:

Datten L. Starmer. PhD, FEACE
Kylie Russell. PhD, SFHEA
Dianne Juliff. PhD

Contact:

Associate Professor Darren Starmer
Head of Assessment
School of Medicine (Fremantle)
The University of Notre Dame Australia
PO Box 1225
Fremantle WA 6959
Australia

Second Edition July 2021

The framework has been developed as a result of intensive consultation with medical practitioners and education consultants in Australia, in review of the Cancer Council Australia's Ideal Oncology Curriculum for Medical Schools:

> Oncology Education Committee. Ideal Oncology Curriculum for Medical Schools [Electronic document]. Sydney: The Cancer Council Australia; 2007 [Available from: https://wiki.cancer.org.au/oncologyformedicalstudents/Ideal_Oncology_Curriculum]

The images used in the graphics contained within the framework are royalty free stock images sourced from Pixabay. https://pixabay.com/service/license/

Introduction

This framework has been designed to assist medical schools to incorporate important components of cancer education into an existing medical curriculum. The framework aims to provide a minimal set of clinical experiences and learning outcomes, which if resourced, will provide Australian medical students with a basic understanding of the knowledge and principles underpinning current cancer management.

Background

The impact of cancer on the Australian population and health care system cannot be overstated. Currently, half of all Australians will be diagnosed with a cancer by the age of 85 (1, 2). Whilst cancer is the leading cause of death in Australia, more than two-thirds of patients are alive five years after their diagnosis (3). Consequentially, there are more Australians living with cancer, placing an incredible demand on the health system (4). Several studies have shown that Australian medical students are ill-prepared to care for cancer patients upon graduation (5-8). Additionally, medical students and junior doctors themselves have highlighted shortcomings in their own cancer education (8-12). The lack of a national medical curriculum results in individual medical schools with considerable diversity in teaching and clinical exposure (5, 13). As such, no minimum requirements for cancer education exist in Australia. At a national level, an ideal oncology curriculum (14, 15) and a palliative care curriculum have been developed (16). To date, it is unclear as to the level of uptake of either curricula within Australian medical schools (8, 10). There remains a lack of consensus on what content to include in a cancer curriculum (17, 18) and how best to deliver such a curriculum (19). In panel sessions conducted with Australian cancer clinicians reviewing the Ideal Oncology Curriculum for Medical Schools (15), it was agreed that medical students require a fundamental understanding of the principles of cancer management, coupled with exposure to cancer patients in cancer service units, in order to observe patient care in the clinical setting. Similarly, there was agreement that medical students do not require specialist knowledge, such as drug or radiotherapy dosages. These findings are consistent with those presented in both national and international literature (20-25).

A Simplistic Approach

The acquisition of the basic principles of cancer management has provided the basis for the development of the Cancer Education Framework for Australian Medical Schools. Once the salient points of management were identified, the necessary knowledge required to underpin these points were identified and incorporated into the framework. Knowledge that would be expected in all Australian medical schools (such as the cell cycle, concepts of incidence and mortality and evidence-based practice) have not been included in the framework. The rationale here is to keep the framework as simple as possible to optimise its utility and adoption by Australian medical schools. The learning objectives presented in the framework are by no means exhaustive but rather provide a minimal blueprint from which to build a basic cancer curriculum within an existing medical school curriculum. The framework draws heavily from the Cancer Council Australia's Ideal Oncology Curriculum for Medical Schools (15), and the aforementioned review by cancer clinicians (26). Other curricula used in the development of this framework include the Palliative Care Curriculum for Undergraduates (16) and the International Summer School 'Oncology for Medical Students' curriculum (25).

International Applicability

Cancer is a global concern and a review of the framework by cancer clinicians and general practitioners from Europe and North America highlighted the applicability of the framework to international medical school curricula. The **local context** section of the framework could easily be modified to incorporate the incidence and mortality, and cancer disparities, specific to the country in which the medical school is situated. This could be further modified to address any additional regional issues as required.

The Framework

The framework is comprised of three sections: one focusing on clinical exposure to cancer patients and clinical cancer service units, whilst the other two focus on the principles of cancer management and cancer-specific knowledge:

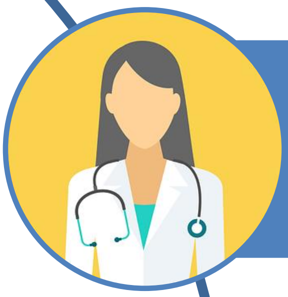

Clinical Exposure

- The five essential cancer clinical experiences

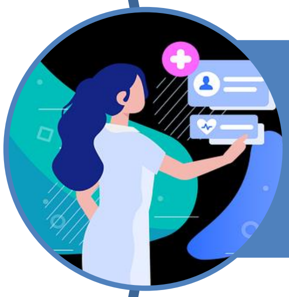

Principles of Cancer Management

- Principles of Surgery
- Principles of Medical Oncology
- Principles of Radiation Oncology
- Principles of Palliative Care

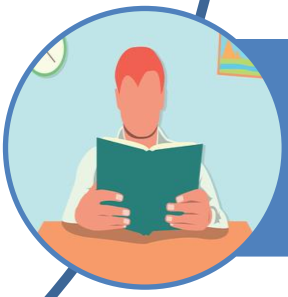

Cancer-specific Knowledge

- Local Context
- Cancer Prevention
- Cancer Biology
- Diagnostic Process
- Patient Centre Focus

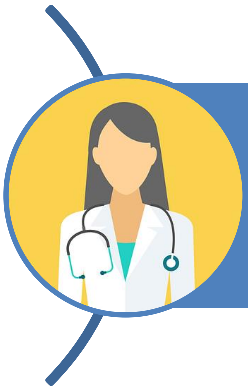

Clinical Exposure

The five essential cancer clinical experiences

A cancer curriculum should include clinical placements that provide medical students with the five clinical cancer experiences, as outlined in the Cancer Council Australia's Ideal Oncology Curriculum for Medical Schools (15, p.45):

- Talking with and examining people affected by all stages of cancer
- Talking with and examining people affected by all common cancers
- Observing all components of multidisciplinary cancer care
- Seeing shared decision-making between people with cancer and their doctors
- Talking with and examining dying people

Exposure to cancer service units

It is recommended that clinical exposure be provided, where possible, through placements in cancer service units, including medical and radiation oncology, and palliative care.

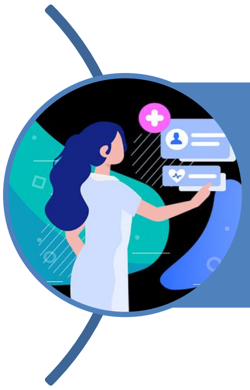

Principles of Cancer Management

General Principles of Cancer Management

Upon graduation, students should be able to:

- Discuss the difference between treatment approaches with curative and palliative intent
- Outline the principles of multidisciplinary management
- Discuss how tumour and patient factors influence the way in which patients are managed
- Describe the role of neo-adjuvant and adjuvant therapy
- Outline the roles of locoregional and systemic therapy
- Outline organ-sparing approaches
- List common oncological emergencies and outline how these are managed
- Discuss the principles of symptom control
- Discuss the role of clinical practice guidelines
- Discuss the role of clinical trials

Principles of Surgery

Upon graduation, students should be able to:

- Outline the aim of cancer surgery
- Describe the importance of adequate surgical margins
- Discuss general preoperative factors
- Identify common complications of cancer surgery and how these can be managed
- Discuss the risks of tumour spill
- Describe the role of surgery in tumour staging

Principles of Radiation Oncology

Upon graduation, students should be able to:

- Outline the aim of radiotherapy
- Discuss indications for radiotherapy
- Describe the cellular response to radiotherapy
- Identify the various methods used to deliver radiotherapy
- Discuss why radiotherapy is delivered using fractionated doses
- Identify common side effects of radiotherapy and how they are managed

Principles of Medical Oncology

Upon graduation, students should be able to:

- Outline the role of medical oncology
- Discuss indications for systemic therapies
- Describe the method of action of systemic agents (i.e. chemotherapy, targeted and hormonal therapies and immunotherapies)
- Identify common side effects of systemic therapy and how they are managed

Principles of Palliative Care

Upon graduation, students should be able to:

- Outline the role of palliative care
- Discuss common end of life issues
- Discuss the provision of palliative care in various settings
- Discuss the role of other modalities in the palliative setting
- Identify commonly used procedures to relieve symptoms
- Outline the role of the GP in providing palliative care

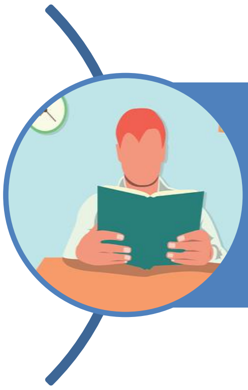

Cancer-specific Knowledge

Local context

Upon graduation, students should be able to:

- Identify the most commonly occurring cancers in men and women in Australia
- Identify the leading causes of cancer death in men and women in Australia
- Outline the differences in cancer outcomes between Indigenous[1] and non-indigenous Australians
- Outline the differences in cancer outcomes between urban and rural Australians

Cancer prevention

Upon graduation, students should be able to:

- Describe methods of primary and secondary prevention
- Differentiate between population-based screening and surveillance
- Discuss the scientific evidence to support population-based methods of screening for cancer
- Identify risk factors for common cancers

Cancer biology

Upon graduation, students should be able to:

- Outline the concept of carcinogenesis
- Describe dysplasia, carcinoma in situ, invasive cancer
- Describe tumour types
- Identify important familial cancer syndromes
- Discuss hormonal influences and tumour markers for common cancers
- Describe patterns of spread of common cancers
- Describe recurrence patterns of common cancers

[1] In Australia Aboriginal and Torres Strait Islander people are acknowledged as the original inhabitants. No disrespect is intended to the Aboriginal and Torres Strait Islander peoples through the use of the internationally used term Indigenous.

Diagnostic process

Upon graduation, students should be able to:

- Outline the necessity of a histopathological diagnosis
- Discuss staging and grading of tumours
- Describe the prognostic implications of differentiation
- Identify potential cancer presentations
- Describe the physical signs of cancer
- Discuss commonly used diagnostic investigations

Patient-centred care

Upon graduation, students should be able to:

- Discuss the importance of involving patients in the decision making process
- Identify factors that influence patient choices
- Outline the impact of bad news on the patient’s ability to process information
- Discuss the psychological impact of screening and diagnostic tests
- Consider the patient's own social context and how a cancer diagnosis will affect the patient, their family and carers
- Discuss the role of cancer support groups
- Outline the role of health professionals in survivorship care
- Identify reliable and accurate sources of information for patients

Resources

There are a number of resources that can be used to underpin the learning objectives presented in the framework, which relate to cancer-specific knowledge and the principles of cancer management. In many instances, medical schools will already have suitable resources, or will wish to create their own. Some schools may wish to incorporate external resources or simply provide the framework to students as an extracurricular learning opportunity. Given that the five essential cancer clinical experiences represent experiential learning, these will require resourcing by the school. To assist schools and students in addressing the learning outcomes comprising the knowledge components of the framework, some suggested resources are included below.

It should be noted that none of these resources are exactly matched to the learning outcomes in the framework. In many cases, they cover content at a greater depth than required, or extend beyond the scope of the framework. However, these resources provide a good starting point and are freely available on the Internet, making them accessible to all schools and students.

Ideal Oncology Curriculum for Medical Schools (IOC) was published by Cancer Council Australia (CCA) and used as the basis for the development of the Cancer Education Framework for Australian Medical Schools:

https://wiki.cancer.org.au/oncologyformedicalstudents/Ideal_Oncology_Curriculum

Clinical Oncology for Medical Students, is an e-book produced by CCA as a resource to support the IOC:

https://wiki.cancer.org.au/oncologyformedicalstudents/Clinical_Oncology_for_Medical_Students

Palliative Care Curriculum for Undergraduates (PCC4U) is a joint collaboration between Queensland University of Technology, the Queensland Government, Flinders University and Curtin University of Technology, with funding provided by the Australian Government Department of Health. The PCC4U curriculum provides a number of resources including several online modules:

https://pcc4u.org.au/learning/modules-landing/

Oncology for Medical Students is one of several YouTube channels covering cancer-related topics. This channel has 26 videos that cover many of the basic principles outlined in the framework:

https://www.youtube.com/channel/UC405maOsEr7erAVgZGDKsJg

Cancer Concepts: A Guidebook for the Non-oncologist as an e-book produced by the University of Massachusetts Medical School. Relevant chapters can be downloaded and contain multimedia and questions.

https://escholarship.umassmed.edu/cancer_concepts/

Learn Oncology is a Canadian web-based resource developed to underpin the Canadian Oncology Goals and Objectives for Medical Students, which was written by the Canadian Oncology Education Group. The web site contains learning modules, videos, virtual patients and quizzes.

https://www.learnoncology.ca/

References

1. Cancer Australia. Cancer in Australia statistics Sydney: Cancer Australia; 2019 [Available from: https://canceraustralia.gov.au/affected-cancer/what-cancer/cancer-australia-statistics].

2. Australian institute of Health and Welfare. Australia's health 2018. Canberra: AIHW; 2018. Contract No.: AUS 221.

3. Arnold M, Rutherford MJ, Bardot A, Ferlay J, Andersson TML, Myklebust TÅ, et al. Progress in cancer survival, mortality, and incidence in seven high-income countries 1995–2014 (ICBP SURVMARK-2): a population-based study. The Lancet Oncology. 2019;20(11):1493-505.

4. Australian Institute of Health and Welfare. Health system expenditure on cancer and other neoplasms in Australia: 2008–09. Canberra: AIHW; 2013.

5. Barton MB, Tattersall MH, Butow PN, Crossing S, Jamrozik K, Jalaludin B, et al. Cancer knowledge and skills of interns in Australia and New Zealand in 2001: comparison with 1990, and between course types. The Medical Journal of Australia. 2003;178(6):285-9.

6. Starmer DL, Barton MB. Advances in cancer management: at what cost to medical student education? J Cancer Educ. 2009;24(3):233-7.

7. Smith WT, Tattersall MH, Irwig L, Langlands AO. Undergraduate education about cancer. European Journal of Cancer. 1991;27(11):1448-53.

8. Starmer DL, House CL, Langworthy KM. Student Exposure to Cancer Patients: an Analysis of Clinical Logbooks and Focus Groups in Clinical Year Medical Students. J Cancer Educ. 2019;35(4):760-5.

9. Bravery B. Cancer survivor and medical student urges better oncology education for doctors: ABC; 2017 [Available from: http://www.abc.net.au/news/health/2017-03-04/changing-oncology-education-for-the-better/8323380].

10. Georgiou G. The changing face of cancer in Australian medical schools. Aust Med Stud J. 2017;8(1):53-6.

11. Langworthy K. A Junior Doctor's Perspective on Oncology and Palliative Medicine Education in Western Australia: Comparison Between Graduation and Completion of Internship. J Cancer Educ. 2019.

12. McRae RJ. Oncology Education in Medical Schools: Towards an Approach that Reflects Australia's Health Care Needs. J Cancer Educ. 2016;31(4):621-5.

13. Starmer DL, Chapman E, Millward MJ. Applying global frameworks to assessment in medical education: an example of a nationally produced curriculum for cancer education. J Cancer Educ. 2010;25(3):285-9.

14. Oncology Education Committee. Ideal Oncology Curriculum for Medical Schools. Sydney: The Cancer Council Australia; 1999.

15. Oncology Education Committee. Ideal Oncology Curriculum for Medical Schools. Sydney: The Cancer Council Australia; 2007.

16. Palliative Care Curriculum for Undergraduates (PCC4U) Project Team, editor. PCC4U Implementation Guide. Brisbane: QUT; 2014.

17. Cancer Council Australia and the Clinical Oncological Society of Australia. Submission to the Department of Education, Science and Training study of medical education in Australia. 2006.

18. Koczwara B, Barton MB, Olver IN, Tattersall MH, Turner DR, Starmer DL. How not to effect change in curricula [letter]. Med J Aust. 2006;185(1):52.

19. Gaffan J, Dacre J, Jones A. Educating Undergraduate Medical Students About Oncology: A Literature Review. Journal of Clinical Oncology. 2006;24:1932-9.

20. Barton MB, Bell P, Sabesan S, Koczwara B. What should doctors know about cancer? Undergraduate medical education from a societal perspective. The Lancet Oncology. 2006;7:596-601.

21. de Vries J. Essentials in Cancer Education. J Cancer Educ. 1999;14(4):198-202.

22. Haagedoorn EM, de Vries J, Robinson E. The UICC/WHO-CCCE Cancer Education project: a different approach. J Cancer Educ. 2000;15(4):204-8.

23. Haagedoorn EM, Oldhoff J, Bender W, Clarke WD, Sleijfer DT. Essential Oncology for Health Professionals. Assen: Van Gorcum & Comp; 1994. 405 p.

24. Junqueira AC. Medical student cancer education: experience from projects in Sao Paulo, Brazil, and in other parts of Latin America. Journal of Surgical Oncology. 2001;77(1):1-4.

25. WHO Collaborating Centre for Cancer Education, Groningen University Faculty of Medical Sciences. 8th International Summer School 'Oncology for Medical Students' Groningen, The Netherlands, 18-28 July 2006. 2006.

26. Starmer DL, Chapman E, Millward MJ. Using expert panels to determine the level of cancer knowledge required of junior doctors in Australia. Part 1: methodology and results. J Cancer Educ. 2013;28(1):60-5.

[This page intentionally left blank]

CANCER EDUCATION FRAMEWORK FOR AUSTRALIAN MEDICAL SCHOOLS

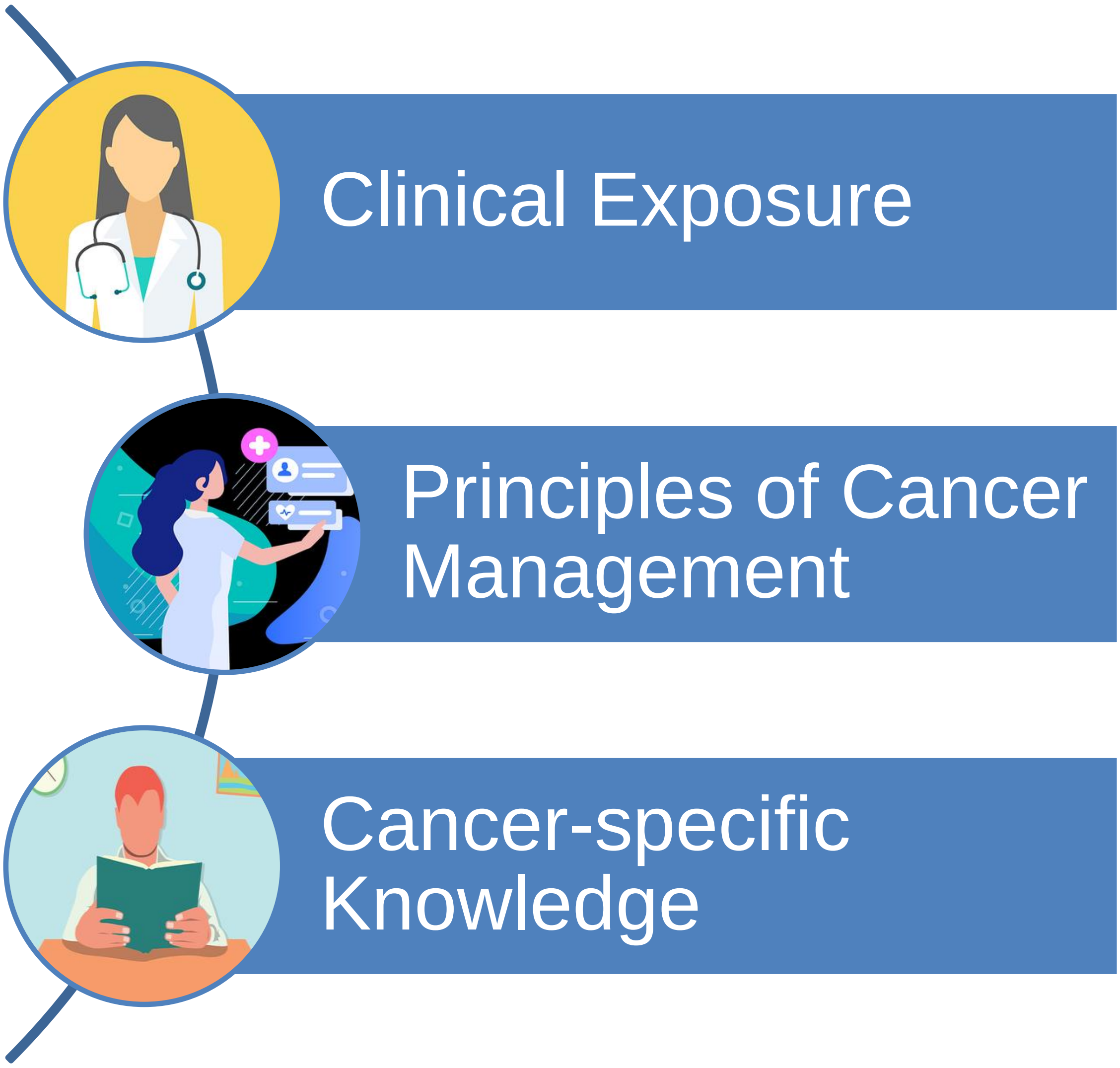

Supplement: Supplementary file 1 — Supplementary file1 (PDF 481 KB) [file 13187_2022_2173_MOESM1_ESM.pdf]
